# A Feasibility Randomized Controlled Trial of Nurse-Led Screening-Based Early Care Program from the Time of Diagnosis for Patients with Advanced Lung Cancer and Their Caregivers

**DOI:** 10.1089/pmr.2025.0018

**Published:** 2025-05-08

**Authors:** Takashi Sato, Hiroya Manaka, Miyuki Kodama, Rina Nieda, Yasumi Kawamura, Mina Yamamoto, Yasuko Genkai, Yumiko Kezuka, Atsuko Watanabe, Nobuki Kaizuka, Hiroki Ito, Hideyuki Sone, Masayuki Shirasawa, Seiichiro Kusuhara, Mikiko Kakegawa, Yoshiro Nakahara, Daisuke Fujisawa, Jiichiro Sasaki, Katsuhiko Naoki

**Affiliations:** ^1^ Department of Respiratory Medicine, Kitasato University School of Medicine, Sagamihara, Japan.; ^2^ Department of Nursing, Kitasato University Hospital, Sagamihara, Japan.; ^3^ Kitasato University Graduate School of Nursing, Sagamihara, Japan.; ^4^ Division of Patient Safety, Keio University School of Medicine, Tokyo, Japan.; ^5^ Department of Neuropsychiatry, Keio University School of Medicine, Tokyo, Japan.; ^6^ Research and Development Center for New Medical Frontiers, Kitasato University School of Medicine, Sagamihara, Japan.

**Keywords:** advanced cancer, early palliative care, family caregiver, feasibility, lung cancer, randomized controlled trial

## Abstract

**Background:**

Although early palliative care (EPC) integrated into standard cancer care improves the quality of lives of patients with cancer and their caregivers, implementation strategies for EPC programs in individual clinical settings have not been established.

**Objective:**

This pilot randomized controlled trial explored the feasibility, acceptability, and effectiveness of nurse-led EPC using a screening-based program that focused on the individual concerns of patients with advanced lung cancer and their caregivers.

**Design:**

This pilot study was a parallel-group randomized controlled trial in which patients were randomly assigned (1:1) to receive either EPC using the care program or standard care alone.

**Setting/Subjects:**

At one university hospital in Japan, 32 patients with newly diagnosed stage IV lung cancer and their 27 caregivers participated.

**Measurements:**

Feasibility was evaluated using recruitment and interview execution rates. Acceptability and effectiveness of the care program were also evaluated.

**Results:**

The recruitment rate was 91%. The interview execution rates were 14/14 (100%) at baseline, 11/14 (79%) at 1 month, and 12/14 (86%) at 3 months for patients, and 10/11 (91%) at baseline, 6/11 (55%) at 1 month, and 7/11 (64%) at 3 months for caregivers. At 5 months, 55% of patients in the intervention group responded that the delivered care was valuable, while 88% of caregivers in the same group answered that it was valuable. The prevalence of severe concerns in patients and caregivers in the intervention group decreased after 1 month.

**Conclusions:**

This pilot randomized controlled trial revealed that EPC using a screening-based program that focused on individual concerns in patients with advanced lung cancer and their caregivers was feasible and acceptable.

## Key Message

This pilot randomized controlled trial for early palliative care implementation in Japan revealed that a nurse-led early dyadic care program that focused on individual concerns in patients with advanced lung cancer and their caregivers was feasible and acceptable.

## Introduction

Patients with advanced cancer experience various physical, psychosocial, and practical issues.^1,2^ In assisting patients with symptom management, emotional support, personal care, finance, transportation, and communication along with health care professionals, family caregivers also experience physical burden and psychological distress; therefore, they are sometimes called “the second patients.”^2–5^

The delivery of palliative care from the early stages of cancer combined with standard oncology care (early palliative care: EPC) improves the physical/psychological symptoms and quality of life (QOL) of patients^6–9^; however, the results have been inconsistent.^10–12^ A randomized controlled trial in Japan did not demonstrate significant effects of a nurse-led specialized EPC intervention program.^13^ Additionally, routine provision of specialized EPC in real-world clinical settings is not practical as it requires plentiful medical resources. EPC implementation is also dependent on the medical system and sociocultural factors. In Japan, although the government’s Basic Plan to Promote Cancer Control promotes the provision of palliative care beginning from cancer diagnosis as an essential component of care for patients, an effective EPC delivery model has not been established primarily due to limited medical resources for specialized palliative care. Meanwhile, physicians, oncologists, and nurses who are not necessarily palliative care experts have been learning and implementing palliative care in clinical practice.

EPC may also improve the outcomes of family caregivers of patients with advanced cancer. McDonald et al. demonstrated that compared with those receiving standard oncology care only, caregivers of patients receiving EPC intervention had greater satisfaction with care but not QOL.^14^ Dionne-Odom et al. showed that EPC intervention on family caregivers through telephone coaching lessened their depression and stress burden compared with delayed intervention, although there was no significant difference in QOL^15^ Nevertheless, how to approach and involve family caregivers has remained a challenge^16^ and is also dependent on sociocultural situations and medical systems.

Thus, we created a screening/interview-based early care program for EPC implementation in clinical practice for both patients and caregivers. Previously, multifaceted concerns in both patients with advanced cancer and their caregivers have significant impact on their psychological distress at diagnosis, and some concerns persisted after diagnosis despite the patients and their caregivers receiving regular follow-ups.^2^ The authors hypothesized that identifying and addressing these concerns from the early stages of cancer treatment would alleviate distress in patients and their caregivers, thereby increasing QOL. Accordingly, we developed a novel model of EPC intervention, in which, based on the systematic screening of the concerns of patients and caregivers using our checklist, our team nurses counseled them with a comprehensive assessment and conducted appropriate care coordination from the time of diagnosis. In this study, a randomized controlled trial was designed to primarily investigate the feasibility and secondarily explore the acceptability and effectiveness of the proposed EPC model.

## Methods

### Study design

This pilot study was a parallel-group randomized controlled trial. Considering their emotional state, participants were approached in clinic as soon as possible after being diagnosed with stage IV lung cancer. After providing written consent, participants were randomly assigned to the intervention and control groups in a 1:1 ratio. Allocation was stratified by (i) age (<70 years or ≥70 years), (ii) histology (nonsmall or small cell lung carcinoma), and (iii) enrollment of corresponding caregivers. Participants were asked to complete the questionnaires, including health-related QOL and mood status, after submitting written informed consent 1, 3, and 5 months later. This study was approved by the institutional review board of Kitasato University Hospital (approval number: C23-040) and registered in the Japanese Clinical Trial Registry (registry ID: UMIN000051704). All participants provided written informed consent.

### Study participants

Patients were eligible if they were (i) newly diagnosed with clinical stage IV lung cancer (8th edition of lung cancer stage classification), (ii) aged ≥20 years, and (iii) able to write and comprehend Japanese. Patients were excluded if they (i) had significant cognitive impairment or (ii) had already received treatment for lung cancer, including chemotherapy, radiation, surgery, or immunotherapy, except for palliative and supportive care. Caregivers were eligible for inclusion if they were (i) identified as the primary family caregivers, (ii) aged ≥20 years, and (iii) able to write and comprehend Japanese. Family caregivers were excluded if they had significant cognitive impairment. Sample size was justified so that if the recruitment rate was ≥75%, lower bound of the 95% confidence interval would be ≥60%.

### Interventions

#### Intervention Group

The intervention group received a screening/interview-based early dyadic care program that focused on individual issues in addition to standard oncology care. A unit of the program consisted of screening of the issues/concerns via a questionnaire and counseling interviews and care coordination based on the screening by team nurses. These procedures were scheduled after enrollment and 1 and 3 months later.

#### Control Group

The control group received standard oncology care in our hospital. It included timely counseling by nurses and/or members in the support center as needed: for example, when patients and/or caregivers consulted. Additionally, patients’ pain was screened using the questionnaire on ease of living^17^ at the time of hospitalization. According to the screening, medical professionals could consult the specialized palliative care team. General palliative care was also provided by physicians, oncologists, and/or nurses.

#### Screening

Both patients and caregivers in the intervention group were requested to complete a brief screening questionnaire, namely the modified “Concerns Checklist.”^2,18^ The Concerns Checklist includes 14 items on various concerns that were not limited to physical issues. The checklist items included the following: C1: your physical symptoms; C2: knowledge or information about illness or treatment (e.g., standard treatment, genome medicine); C3: anything to do with treatment or care (e.g., side effects); C4: not being able to do the things you usually do (e.g., social activities); C5: caring for yourself (e.g., eating, bathing, movement, excretion); C6: lack of care or support; C7: worries or concerns about important people in your life (e.g., partners, children, family); C8: communications with health care professionals or people around you; C9: your finances; C10: your work; C11: mental/spiritual issues; C12: how to deal and cope with illness; C13: discussions on end-of-life care; and C14: any other concerns you consider important (free-format). Using a scale ranging from 0 to 3 (0: “not at all,” 1: “a little,” 2: “quite a bit,” and 3: “very much”), participants rated how much each item (C1–C13) existed during the previous week. In this study, we defined a patient and/or a family caregiver as having a “serious” concern if they scored ≥2 on any of the items.

#### Assessment, Counseling, and Care Coordination by Team Nurses

Nurses assigned to our respiratory or oncology department, who were not limited to certified nurse specialists or certified nurses interviewed the patient and his/her caregiver and conducted a comprehensive assessment using the Concerns Checklist. Nurses focused on and inquired about the “serious” concerns of patients and their caregivers. Additionally, nurses attempted to reveal whether there were hidden concerns, and they focused not only on the concerns but also on the strengths of patients and their caregivers to help them cope better with the illness. Based on the concerns and strengths, nurses attempted to provide the following care as needed: building rapport, symptom management, facilitating coping with cancer, facilitating illness understanding, facilitating advance care planning, share decision-making on treatment or disposition, and helping communication with medical professionals and/or within family. Nurses help patients and caregivers improve their situation and physical/mental status by providing counseling themselves or by coordinating referrals to other professionals as needed. Information on the extracted concerns and requests was shared with attending physicians, nurses in charge, and care team members. For example, symptom management might be addressed through advising how to take pain medications, sharing the information with attending physicians and/or referring to specialized palliative care; financial concerns through providing information by themselves and/or referring to social workers.

Before starting these interventions, the research nurses underwent a training/instruction on the care program described above in our team meeting for approximately 30 minutes. There was no special training program required since our care program was an extended model from daily oncology and palliative care delivered by regular medical professionals. The nurses who had been learning and implementing palliative care in our clinical practice and were not necessarily palliative care specialists demonstrated their skills. The investigators regularly monitored the intervention fidelity through the interview records written by the nurses in a specific format.

### Measurements

#### Feasibility

The primary objective was to explore the feasibility of the intervention as determined by recruitment and interview execution rates in the intervention group. The recruitment rate was the percentage of enrolled participants from the total number of eligible patients. The interview execution rate was the percentage of interviews from the total number of planned interviews at baseline, 1 month, and 3 months. Our protocol was assumed to be feasible if the recruitment rate was ≥75% and the interview execution rates at baseline and 1 and 3 months were ≥80% and ≥50%, respectively.

The collection rate of the questionnaires was the percentage of participants who submitted completed questionnaires over the number of participants scheduled for follow-up at each time point.

#### Acceptability

Using a scale ranging from 1 to 4 (1: “not valuable at all,” 2: “not very valuable,” 3: “a little valuable,” and 4: “very valuable”), patients and caregivers in the intervention group rated how much the screening/interview-based care was valuable at 5 months. They were also asked to select helpful elements of the care from the following options: alleviation of patient’s physical symptoms; understanding of illness or treatment; how to spend daily life; how to receive care or support; worries or concerns about important people in your life; communications with health care professionals; financial issues; work issues; mental/spiritual issues; how to deal and cope with illness; discussions on end-of-life care; alleviation of caregiver’s physical symptoms (caregivers only). Multiple answers were allowed. Additionally, nurses who interviewed the participants also rated how valuable the screening/interview-based care was using the same scale after each interview. For patients or caregivers, the acceptability rate was the percentage of participants that responded that the interventions were very or a little valuable from the total number of them. Our protocol was assumed to be acceptable if the acceptability rate was ≥50%.

#### QOL

Health-related QOL of patients was measured using the Functional Assessment of Cancer Therapy–Lung (FACT-L) scale, which measures multiple dimensions of QOL (physical, social, emotional, and functional well-being) of patients with lung cancer and lung cancer symptom burden during the past week.^19,20^ Health-related QOL of caregivers was measured using the Caregiver Quality of Life Index-Cancer (CQOLC), which consists of four domains: psychological burden (eight items); positive emotions (five items); financial burden (three items); and disruption of daily living (five items).^21,22^ In both scales, higher scores indicated better QOL. Scores were measured at baseline and 1, 3, and 5 months, and the change from baseline to each point for each score was calculated as a secondary outcome in this study.

#### Mood Status

Mood symptoms of patients and caregivers were assessed using the Hospital Anxiety and Depression Scale (HADS), which is a 14-item self-report questionnaire that contains two subscales that assess anxiety and depression^23,24^; higher scores indicate worse symptoms. Mood was assessed at baseline and 1, 3, and 5 months, and the change from baseline to each time point on each subscale score was calculated as a secondary outcome in this study.

### Statistical analyses

After descriptive analyses, bivariate analyses were performed to compare the variables between the two groups. Fisher’s exact test was used for categorical variables, and the Mann–Whitney *U* test was for continuous and ordinal variables. For the Mann–Whitney *U* test, an effect size *r* was calculated by dividing the absolute standardized test statistic *z* by the square root of the number of pairs. All *p* values were two-sided, and *p* < 0.05 was considered statistically significant. Since the sample size is small in this study, *p* values were calculated as reference values. All statistical analyses were performed using SPSS software (SPSS; IBM Corp., Armonk, NY, USA).

## Results

### Participant recruitment

**FIG. 1.**
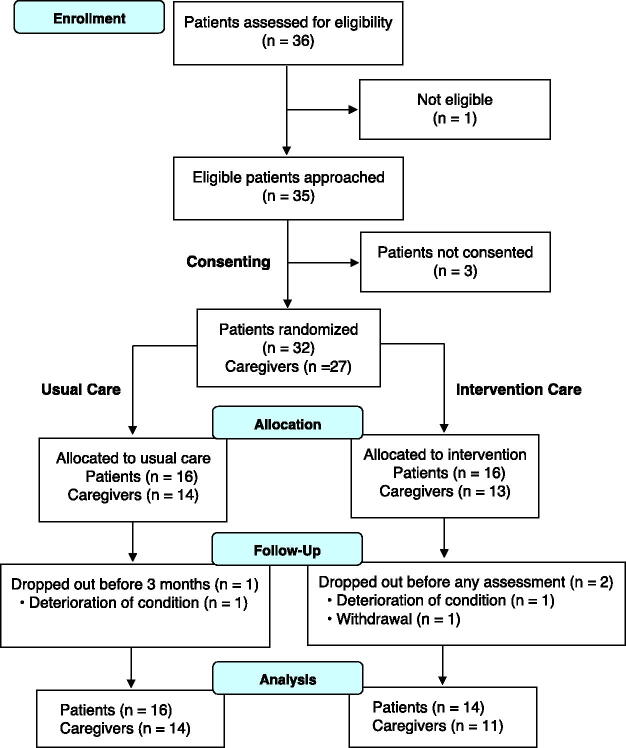
The participants flow diagram.

The flow diagram of the study is shown in Figure 1. From October 4, 2023, to April 24, 2024, 35 patients were approached, with 32 (91%) providing written informed consent. Meanwhile, 27 caregivers (84% of the patients) provided written informed consent to participate. Notably, five patients did not have caregivers as they either did not have a family member or their caregivers were unavailable. Overall, 16 patients and 13 caregivers were allocated to the intervention group, and 16 patients and 14 caregivers were allocated to the control group.

The participants’ characteristics are shown in Table 1. The median age of the patients was 74, 20 out of 32 were male, 25 had nonsmall cell lung cancer, and 7 had small cell lung cancer. Meanwhile, 74% of the participating caregivers were the patients’ spouses. The baseline characteristics were balanced between the two study groups.

**Table 1.** Baseline Characteristics of Study Participants

Patient variable	Control (*n* = 16)	Intervention (*n* = 16)	*p*
Age (year)			
Median	75	72	0.581^a^
Lower-upper quartile	68–76	61–76	
Sex, %			
Male	9 (56)	11 (69)	0.716^b^
Female	7 (44)	5 (31)	
Smoking status (pack year)			
Median	27	40	0.299^a^
Lower-upper quartile	0–46	7–54	
Marital status, %			
Married	13 (81)	10 (72)	0.675^b^
Single/widowed/divorced	3 (19)	4 (28)	
Employment status, %			
Employed	3 (19)	5 (36)	0.417^b^
Unemployed	13 (81)	9 (64)	
ECOG performance status, %			
0	5 (31)	4 (25)	0.867^a^
1	8 (50)	9 (56)	
2	3 (19)	3 (19)	
Histology, %			
Nonsmall cell carcinoma	12 (75)	13 (81)	1.000^b^
Small cell carcinoma	4 (25)	3 (19)	
Clinical Tumor-Node-Metastasis stage, %			
IVA	8 (50)	6 (38)	0.722^b^
IVB	8 (50)	10 (62)	
Initial treatment			
Chemotherapy + ICI	6	5	0.775^b^
Chemotherapy	2	1	
Chemotherapy + molecular targeted therapy	1	0	
ICI	1	2	
ICI + ADC	0	2	
Molecular targeted therapy	2	4	
Whole brain irradiation	1	0	
Undecided	3	2	
FACT-L total score			
Median	74.1	76.4	0.608^a^
Lower-upper quartile	67.0–96.0	59.2–96.5	
HADS-Anxiety			
Median	4	7	0.465^a^
Lower-upper quartile	2–8	3–9	
HADS-Depression			
Median	6	8	0.380^a^
Lower-upper quartile	4–10	5–11	

Caregiver variable	Control (*n* = 14)	Intervention (*n* = 13)	*p*
Age (yr)			
Median	69	69	1.000^a^
Lower-upper quartile	50–75	54–73	
Sex			
Male	6 (43)	3 (25)	0.677^b^
Female	8 (57)	8 (75)	
Marital status			
Married	13 (93)	10 (92)	1.000^b^
Single/widowed/divorced	1 (7)	1 (8)	
Employment status			
Employed	5 (36)	4 (42)	1.000^b^
Unemployed	9 (64)	7 (58)	
Relationship to patient			
Spouse	11 (79)	8 (73)	0.625^b^
Child	4 (21)	2 (18)	
Sibling	0 (0)	1 (9)	
Resides with patient			
Yes	11 (85)	11 (92)	0.482^b^
No	2 (15)	0 (8)	
CQOLC			
Median	62	65	0.695^a^
Lower-upper quartile	54–72	54–79	
HADS-Anxiety			
Median	10	8	0.208^a^
Lower-upper quartile	7–11	5–9	
HADS-Depression			
Median	7	7	1.000^a^
Lower-upper quartile	4–10	2–11	

^a^ Mann–Whitney *U* test.

^b^ Fisher’s exact test.

ADC, antibody–drug conjugate; CQOLC, the Caregiver Quality of Life Index-Cancer; ECOG, Eastern Cooperative Oncology Group; FACT-L, the Functional Assessment of Cancer Therapy–Lung; ICI, immune checkpoint inhibitor; HADS, Hospital Anxiety and Depression Scale.

### Interview execution rates in the intervention group

The interview execution rates at baseline, 1 month, and 3 months for patients and caregivers were 14/14 (100%), 11/14 (79%), and 12/14 (86%), and 10/11 (91%), 6/11 (55%), and 7/11 (64%), respectively.

### Collection rates of the questionnaires

The collection rates at baseline and 1, 3, and 5 months were 30/30 (100%), 28/30 (93%), 28/29 (97%), and 28/29 (97%) for patients, and 25/25 (100%), 23/25 (92%), 23/24 (96%), and 21/24 (86%) for caregivers, respectively.

### Acceptability and perceived value of the interventions

**FIG. 2.**
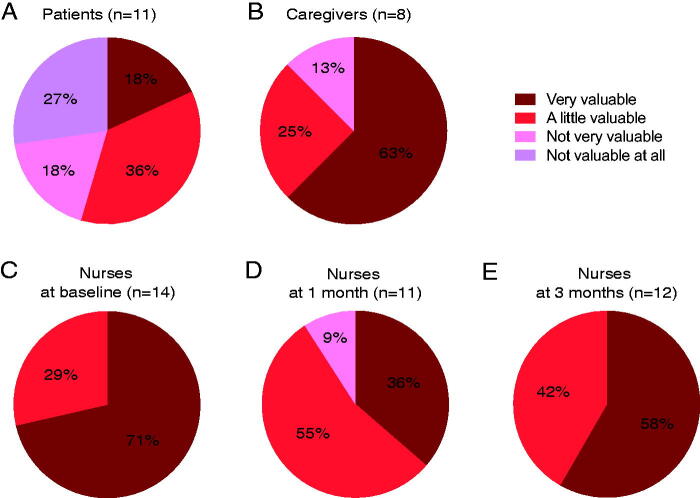
Rating how much the screening/interview-based care in addition to standard care was valuable. **(A)** Rating by patients at 5 months. **(B)** Rating by caregivers at 5 months. **(C–E)** Rating by nurses at baseline **(C)**, 1 month **(D),** and 3 months **(E)**.

Of the 11 patients who completed the questionnaires at 5 months, 2 responded that the interventions were very valuable, 4 responded a little valuable, 2 responded not very valuable, and 3 responded not valuable at all (Fig. 2A). The acceptability rate was 55% for patients.

Of the eight caregivers who completed the questionnaires at 5 months, five responded that the interventions were very valuable, two responded that they were a little valuable, and one responded that they were not very valuable (Fig. 2B). The acceptability rate was 88% for caregivers.

The most helpful elements of the interventions for both patients and caregivers were “understanding of illness or treatment” and “communications with health care professionals” (Supplementary Fig. S1).

Of the 14 interventions at baseline, nurses who interviewed the participants responded that 10 were very valuable, and 4 were a little valuable (Fig. 2C). Of the 11 interventions at 1 month, nurses responded that 4 were very valuable, 6 were a little valuable, and 1 was not very valuable (Fig. 2D). Of the 12 interventions at 3 months, nurses responded that 7 were very valuable, and 5 were a little valuable (Fig. 2E).

Of the 14 interventions at baseline, nurses judged that 1 was very feasible in clinical practice, and 13 were mostly feasible. Of 11 interventions at 1 month, nurses judged that 1 was very feasible in clinical practice, 8 were mostly feasible, and 2 were not very feasible. Of the 12 interventions at 3 months, nurses judged that 8 were mostly feasible in clinical practice, and 4 were not very feasible.

### QOL and mood status

Changes in the FACT-L, HADS-anxiety, and HADS-depression scores at 1, 3, and 5 months in patients in both study groups are shown in Table 2. Although no significant differences were found, the median changes in the FACT-L total score were lower in the intervention group. There were also no significant differences in the changes from baseline in the CQOLC, HADS-anxiety, or HADS-depression scores at 1, 3, and 5 months in caregivers in both study groups (Table 2).

**Table 2.** Change in the Quality of Life and Mood Scores in the Two Study Groups

	Control	Intervention	*r*	*p*
Patient variable				
FACT-L total score				
Median change from baseline to 1 month (1st, 3rd quartile)	4.08 (−5.67, 14.0)	−4.83 (−13.5, 12.3)	0.171	0394
Median change from baseline to 3 months (1st, 3rd quartile)	5.17 (−3.67, 15.0)	−5.67 (−12.0, 10.0)	0.310	0108
Median change from baseline to 5 months (1st, 3rd quartile)	7.50 (−2.00, 17.7)	−1.42 (−10.7, 8.83)	0.226	0246
HADS-Anxiety				
Median change from baseline to 1 month (1st, 3rd quartile)	−0.5 (−2.0, 2.0)	0.0 (−3.0, 2.0)	0.018	0.931
Median change from baseline to 3 months (1st, 3rd quartile)	1.0 (−1.0, 1.0)	0.0 (−2.0, 1.0)	0.157	0.427
Median change from baseline to 5 months (1st, 3rd quartile)	−1.0 (−2.0, 1.0)	−1.0 (−3.0, 0.0)	0.107	0.614
HADS-Depression				
Median change from baseline to 1 month (1st, 3rd quartile)	−1.0 (−2.5, 2.0)	−1.0 (−1.0, 1.0)	0.130	0.512
Median change from baseline to 3 months (1st, 3rd quartile)	0.0 (−2.0, 1.0)	1.0 (−1.0, 1.0)	0.098	0.618
Median change from baseline to 5 months (1st, 3rd quartile)	0.0 (−2.0, 3.0)	0.0 (−3.0, 2.0)	0.127	0.519
Caregiver variable				
CQOLC				
Median change from baseline to 1 month (1st, 3rd quartile)	1.64 (−4.00, 10.0)	8.00 (6.00, 10.0)	0.211	0.336
Median change from baseline to 3 months (1st, 3rd quartile)	7.00 (4.57, 12.0)	4.50 (−5.00, 11.0)	0.201	0.343
Median change from baseline to 5 months (1st, 3rd quartile)	11.0 (6.00, 16.00)	10.0 (0.00, 18.0)	0.031	0.918
HADS-Anxiety				
Median change from baseline to 1 month (1st, 3rd quartile)	−1.5 (−4.0, 0.0)	−1.0 (−4.0, 0.0)	0.060	0.781
Median change from baseline to 3 months (1st, 3rd quartile)	−1.0 (−3.0, 0.0)	−2.0 (−4.0, −0.5)	0.008	0.972
Median change from baseline to 5 months (1st, 3rd quartile)	−3.0 (−5.0, 0.0)	−3.0 (−5.0, 0.0)	0.138	0.552
HADS-Depression				
Median change from baseline to 1 month (1st, 3rd quartile)	1.0 (−2.0, 2.0)	0.0 (−2.0, 0.0)	0.210	0.357
Median change from baseline to 3 months (1st, 3rd quartile)	−1.0 (−2.0, 0.0)	−1.0 (−2.0, 0.0)	0.072	0.744
Median change from baseline to 5 months (1st, 3rd quartile)	−1.0 (−4.0, 1.0)	−1.5 (−2.0, −1.0)	0.047	0.863

CQOLC, the Caregiver Quality of Life Index-Cancer; FACT-L, the Functional Assessment of Cancer Therapy–Lung; HADS, Hospital Anxiety and Depression Scale.

### Trends of concerns over time

**FIG. 3.**
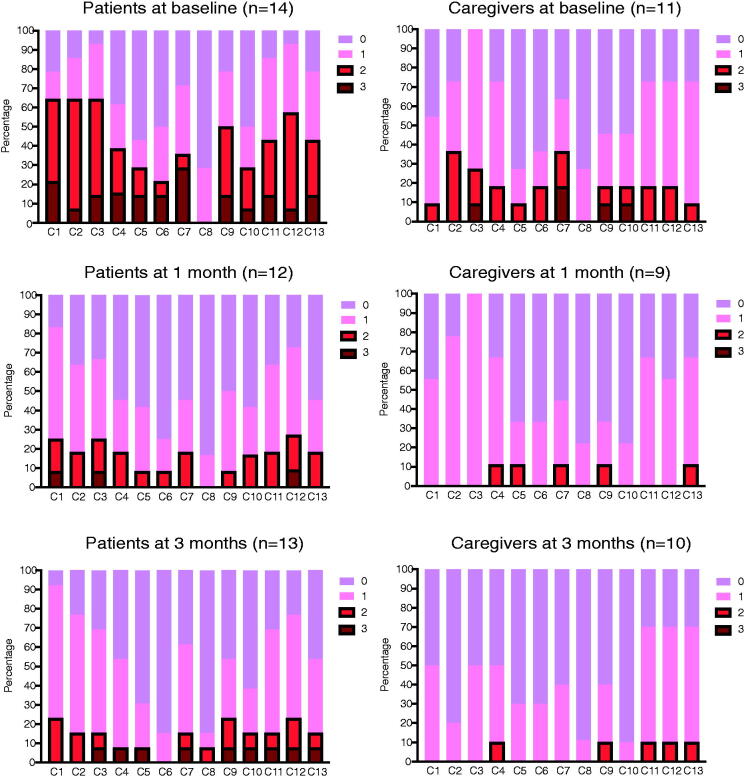
The proportions of the four concern levels in the concern checklist at baseline, 1 month and 3 months in patients and caregivers. Using a scale ranging from 0 to 3 (0: “not at all,” 1: “a little,” 2: “quite a bit,” and 3: “very much”), participants rated how much each item (C1–C13) existed during the previous week. If they scored ≥2 on any of the items, the concern was defined as a “serious” concern. C1: your physical symptoms; C2: knowledge or information about illness or treatment (e.g., standard treatment, genome medicine); C3: anything to do with treatment or care (e.g., side effects); C4: not being able to do the things you usually do (e.g., social activities); C5: caring for yourself (e.g., eating, bathing, movement, excretion); C6: lack of care or support; C7: worries or concerns about important people in your life (e.g., partners, children, family); C8: communications with health care professionals or people around you; C9: your finances; C10: your work; C11: mental/spiritual issues; C12: how to deal and cope with illness; C13: discussions on end-of-life care.

The proportions of the four concern levels in the concern checklist at baseline and 1 and 3 months are presented in Figure 3. After 1 month, the prevalence of the most severe concerns decreased for both patients and caregivers in the intervention group.

### Interview/counseling time and care coordination

Interview/counseling time had an approximate median value of 30 minutes at baseline and 15 minutes at 1 and 3 months (Supplementary Fig. S2). In some cases, total support centers with social workers and nurse consultants were introduced after the interviews.

## Discussion

In this study, a screening/interview-based early dyadic care program focusing on individual issues in addition to standard oncology care was feasible with our criteria for conducting clinical trials and implementing care in clinical practice. Regarding acceptability, our results indicated that most of the patients and caregivers appreciated the care program, and the nurses who conducted the interviews and performed care coordination perceived the value of the intervention.

Implementing EPC and a multifaceted support for patients with cancer and their caregivers in clinical practice is a significant challenge for any medical institution due to the required medical resources, medical systems, and sociocultural factors, including awareness of the importance of EPC in patients, caregivers, and health care professionals.^25^ It was reported that there is very limited utilization of implementation frameworks to integrate EPC into standard oncology practices.^26^ Therefore, we designed a model of intervention and assessment for EPC implementation in clinical practice. We assume that its feasibility, as evaluated by the recruitment and interview execution rates in the intervention group, was sufficient to proceed to the next larger study. The recruitment and retention rates of both 91% in this study were relatively high compared with previous EPC studies that assessed feasibility,^27–31^ in which those rates were 49%–88% and 39%–87%, respectively. A possible reason for this is that our interventions were not highly intensive but seemed beneficial to patients and caregivers so that they tended to willingly participate and continuously receive the interventions in this study. It might be also owing to good relationships between patients/caregivers and medical professionals in our medical and sociocultural environment in Japan. Meanwhile, the modest interview execution rates of 55% and 64% in caregivers at 1 and 3 months, respectively, were primarily due to the difficulty in scheduling meetings with caregivers who were not the direct targets of medical care and did not necessarily visit the hospital with patients. The interview/counseling time in this study was approximately 30 minutes at the first interview, which was longer than that in later interviews (approximately 15 minutes). We assumed that the interview time range was feasible in clinical practice.

Compared with patients, caregivers had a greater tendency to perceive the value of the interventions in addition to standard care in this study. Since caregivers have less chances to directly talk with medical professionals than patients, their concerns are easily overlooked, although the prevalence of concerns among caregivers of patients with advanced cancer is comparable to that among the patients.^2^ Overall, 5/8 caregivers in the intervention group who completed the study protocol chose “communication with medical professionals” as one of the items that had been helpful. In contrast, patients have routine opportunities for medical interviews and screenings when they undergo check-ups, are admitted, and undergo treatment. Therefore, extra interviews and screenings in addition to standard care might be time-consuming or even bothersome, leading to the modest acceptability rate for patients in this study. Nevertheless, more than half of the patients who completed the study protocol responded that the interventions were very/a little valuable. Our interventions may be able to extract individual patient issues that are not addressed by standard care alone, leading to better care coordination. Nurses who interviewed the participants also assessed their interventions highly. Through screenings and interviews, nurses can communicate well with patients and caregivers, identify their problems and strengths, and discuss better strategies for dealing with the disease, which may improve the quality of oncology care.

However, there are barriers and disadvantages to our intervention, the biggest of which is that the interventions are time-consuming and require scheduling by all patients, caregivers, and nurses. Although medical resources are limited in every hospital, all patients and caregivers in the intervention group were scheduled for interviews in this trial. Another pilot trial of EPC highlighted the issues regarding time management of the interventions.^27^ Although every interview is an important opportunity to build or sustain rapport and to reveal hidden concerns and advantages of both patients and caregivers, using the concern checklist or other tools to identify patients and caregivers who truly require interviews and care coordination may conserve medical resources and further improve the acceptability rate for patients by reducing the burden of procedures for them.

Regarding the effectiveness of our interventions, we assessed the self-reported QOL and mood scales of both patients and caregivers, revealing that the interventions did not significantly affect the scores of both scales compared to standard care alone. We assume that it is difficult to detect the differences of the scales with the small sample size of this study. Even though no significant differences of baseline characteristics between both groups were found, we realized that personalities, family relationships, and social situations of the participants were very diverse, which might cause the inconsistency of those scores. Meanwhile, it remains unclear whether the median changes in the total FACT-L scores were somewhat lower in the intervention group. One hypothesis is that although our interventions may assist in patients’ illness understanding, improved understanding may result in a poor perceived QOL.^32,33^ Nevertheless, most of the various concerns of both patients and caregivers in the intervention group were alleviated after 1 month in this study. As many factors, including patient/cancer/caregiver characteristics and care/treatment details, affect patient/caregiver outcomes, a larger and well-designed study is required to validate the effectiveness of the interventions.

This study has some limitations. First, it was conducted in a single university hospital that was relatively well-staffed with medical professionals proficient in cancer care when compared to other hospitals; this may limit its generalizability. However, there is a shortage of medical workforce to manage care for all kinds of patients in our hospital, similar to other hospitals in Japan. Therefore, in this study, pulmonologists and nurses not necessarily specializing in cancer and palliative care attended to the participants, and general nurses not limited to certified nurse specialists or certified nurses interviewed the participants. Second, the small sample size of this pilot trial limited the power to test the effectiveness of the interventions. While a larger-scale randomized control trial may have the potential to test it, some aspects of our interventions such as screening of the patients who really need interventions and timing/intensity of the interventions can be optimized through another pilot study based on the findings in this study.

In conclusion, our pilot randomized controlled trial of nurse-led EPC using a care program focusing on individual concerns of patients with advanced lung cancer and their caregivers was feasible and acceptable for both participants and investigators.

## Notes

**Cite this article as:** Sato T, Manaka H, Kodama M, Nieda R, Kawamura Y, Yamamoto M, Genkai Y, Kezuka Y, Watanabe A, Kaizuka N, Ito H, Sone H, Shirasawa M, Kusuhara S, Kakegawa M, Nakahara Y, Fujisawa D, Sasaki J, Naoki K (2025) A feasibility randomized controlled trial of nurse-led screening-based early care program from the time of diagnosis for patients with advanced lung cancer and their caregivers, *Palliative Medicine Reports* 6:1, 251–262, DOI: 10.1089/pmr.2025.0018.

## Abbreviations Used

ADC: antibody–drug conjugate
CQOLC: the Caregiver Quality of Life Index-Cancer
ECOG: Eastern Cooperative Oncology Group
EPC: early palliative care
FACT-L: the Functional Assessment of Cancer Therapy–Lung
HADS: the Hospital Anxiety and Depression Scale
ICI: immune checkpoint inhibitor
QOL: quality of life
